# FOXM1: a new therapeutic target of extramammary Paget disease

**DOI:** 10.1038/s41598-024-54773-8

**Published:** 2024-02-19

**Authors:** Takamichi Ito, Yuka Tanaka, Yumiko Kaku-Ito, Yoshinao Oda, Takeshi Nakahara

**Affiliations:** 1 Department of Dermatology, Graduate School of Medical Sciences, Kyushu University, 3-1-1 Maidashi, Higashi-ku, Fukuoka, 812-8582 Japan; 2 Department of Anatomic Pathology, Graduate School of Medical Sciences, Kyushu University, Fukuoka, 812-8582 Japan

**Keywords:** FOXM1, Thiostrepton, Cell line, Targeted therapy, Skin cancer, Cancer, Skin cancer

## Abstract

Extramammary Paget disease (EMPD) is a rare skin cancer that primarily affects older individuals predominantly in areas with apocrine sweat glands. Although most early EMPD lesions are indolent, patients with metastatic EMPD have a poor prognosis due to the lack of effective systemic treatment. In this study, we investigated the role of forkhead box M1 (FOXM1), a potent transcription factor, in EMPD and assessed the potential of FOXM1 as a therapeutic target. Immunohistochemistry of 112 primary and 17 metastatic EMPD samples revealed that FOXM1 expression increased with tumor progression. Patients in whom FOXM1 was expressed in more than 10% of tumor cells had significantly shorter disease-specific survival than the other patients (*p* = 0.0397). In in vitro studies using our newly established EMPD cell line, KS-EMPD-1, we found high expression of FOXM1. Knockdown of FOXM1 impaired tumor cell viability, migration, and invasion. Inhibition of FOXM1 using thiostrepton also reduced tumor cell viability in a dose-dependent manner. These findings suggest that FOXM1 is a promising therapeutic target for patients with EMPD.

## Introduction

Extramammary Paget disease (EMPD) is a rare skin cancer that primarily occurs in older individuals predominantly in areas with apocrine sweat glands ^1–6^. The pathogenesis and risk factors of EMPD are not well understood, but it typically affects Caucasian women and Asian men over 60 years old ^2,7–9^. The crude prevalence of EMPD in mainland China was reported to be 0.4 per million people in 2016 and the age-standardized incidence in Europe is 0.6 per million person-years ^10,11^. Most EMPD tumors are slow-growing and remain indolent as in situ lesions for long periods, resulting in a favorable prognosis for patients ^12–14^. It is worth noting that EMPD lesions can sometimes be difficult to diagnose clinically, as they can bear a striking resemblance to other types of inflammatory skin diseases ^2^. Furthermore, it is important to be aware that dermal invasion occurs in a significant proportion of cases (between 15 and 40%), which can increase the likelihood of metastasis ^2,3,15^. Given these potential risks, it is strongly recommended that individuals seek out early surgical removal of EMPD lesions in order to minimize their potential impact on overall health and wellbeing ^6^. It is also important to distinguish EMPD from mammary Paget disease, which is an epidermal extension from breast cancer, and secondary EMPD, which is an extension from visceral cancers ^1,2^. A panel of immunohistochemical staining such as CK7, CK20, GCDFP15, CDX2, and TRPS1 can be helpful in making this distinction ^1,2,15,16^. EMPD lesions often have an unclear tumor border and may have hidden skip lesions ^17^. To completely remove the tumors, various methods have been used, such as wide local excision with mapping biopsies, photodynamic diagnosis-guided surgery, and Mohs micrographic surgery ^18–21^. However, complete removal of the tumors is sometimes challenging due to anatomical constraints ^22^. Conventional chemotherapies with anticancer drugs such as TS-1, docetaxel (DTX), paclitaxel (PTX), 5-fluorouracil (5-FU), or cisplatin (CDDP) are used for metastatic EMPD, but the prognosis for such cases is generally poor ^23–25^; there is thus an urgent need for alternative treatments.

In the pursuit of novel therapeutic targets for EMPD, researchers have primarily concentrated on human epidermal growth factor receptor 2 (HER2), which is encoded by the *ERBB2* gene ^26,27^. Several sporadic cases have demonstrated promising results with HER2 antibody and chemotherapy combinations ^28–30^. Currently, other potential targets are being extensively studied to broaden our understanding of EMPD treatment options, including androgen receptor ^31–34^, programmed death 1/programmed death-ligand 1 ^35–37^, cyclin-dependent kinase 4 ^38^, C-X-C chemokine receptor type 4 (CXCR4), CXCR7 ^39^, phosphatidylinositol 4,5-bisphosphate 3-kinase catalytic subunit alpha (PIK3CA) ^40^, dynamin-related protein 1 (DRP1) ^41^, nectin cell adhesion molecule 4 (NECTIN4) ^42,43^, trophoblast cell surface antigen 2 (TROP2) ^44^, and forkhead box A1 (FOXA1) ^45^. However, the results were mainly based on analyzing formalin-fixed paraffin-embedded (FFPE) or fresh/frozen clinical tissue samples using immunohistochemical or nucleic acid-based approaches. To reveal the detailed pathogenetic mechanisms of EMPD, cellular methods including in vivo or in vitro disease models are required.

Forkhead box M1 (FOXM1) is a transcription factor belonging to the FOX family, which is expressed in embryonic tissues ^46–49^. Its regulated expression and activity play a crucial role in ensuring proper growth and maturation. FOXM1 is also involved in regulating significant processes in tumor development and progression such as proliferation, migration, angiogenesis, and chemoresistance ^46–49^. Previous studies have reported that FOXM1 is overexpressed in various cancers and sarcomas including breast cancer, melanoma, angiosarcoma, lung cancer, pancreatic cancer, gastric cancer, prostate cancer, leukemia, leiomyosarcoma, and synovial sarcoma ^48–59^. The overexpression of FOXM1 is associated with poor patient survival in many of these tumors, in which advanced tumors tend to more strongly express FOXM1. However, the role of FOXM1 expression in EMPD tumorigenesis and tumor progression remains unclear, as little information is available on its expression in EMPD. However, the difficulty in establishing in vivo or in vitro models of EMPD had hampered basic research to explore the pathobiology of EMPD. We recently established a novel EMPD cell line, named KS-EMPD-1 ^60^. In this study, we investigated FOXM1 expression and its role in tumor proliferation and development in EMPD patients’ clinical samples and in this cell line. FOXM1 expression increased with tumor progression in patients’ clinical samples, and high FOXM1 expression in primary tumors was significantly associated with shorter disease-specific survival. In in vitro study using KS-EMPD-1, FOXM1 was associated with tumor cell viability, migration, and invasiveness, and its inhibition strongly impaired tumor cell viability.

## Results

### FOXM1 expression in EMPD patient tissue

**Figure 1:**
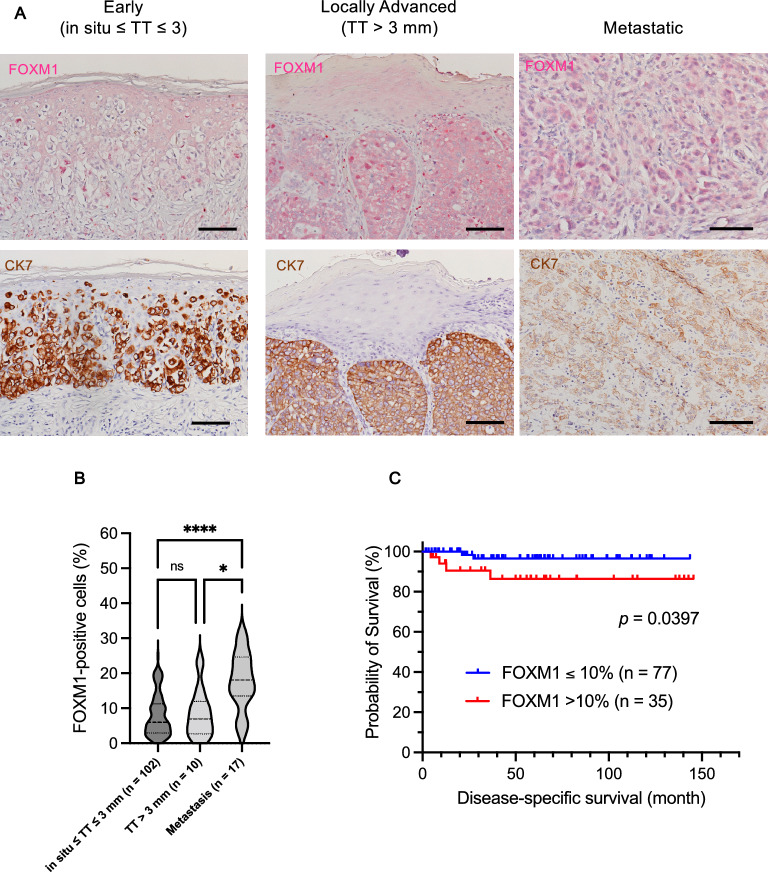
FOXM1 expression in patients’ EMPD tissues. (**A**) Immunohistochemical images of FOXM1 in patients’ EMPD tissues. Representative images of early lesions [tumor thickness (TT) ≤ 3 mm, n = 102], locally advanced lesions (TT > 3 mm, n = 10), and metastatic lesions (n = 17) are shown. Scale bars = 100 μm. (**B**) Comparison of FOXM1-positive cells (%) among early, locally advanced, and metastatic lesions of patients’ tissues. *p < 0.05 and ***p < 0.001. (**C**) Survival curves of patients with ≤ 10% (n = 77) or > 10% (n = 35) FOXM1 staining positivity in immunohistochemistry. Patients with FOXM1 > 10% had significantly shorter disease-specific survival than those with FOXM1 ≤ 10% (*p* = 0.0397).

To investigate the expression of FOXM1 in EMPD lesions, 112 samples of primary EMPD and 17 samples of metastatic disease were immunohistochemically stained for FOXM1 and observed. Demographic and clinical data of the patients are listed in Table 1. As shown in Fig. 1A, FOXM1 was expressed in most of the EMPD samples, at least in part. FOXM1-positive cells increased in number with tumor progression; early lesions had fewer positive cells, while metastatic lesions had more of them (*p < 0.05 and ***p < 0.001, Fig. 1B). To further investigate the impact of FOXM1 expression level on patient survival, the patients were categorized into two groups (FOXM1 ≤ 10% and FOXM1 > 10%), and the relationship between FOXM1 expression and survival was analyzed. Patients with FOXM1 > 10% had significantly shorter survival than those with FOXM1 ≤ 10% (*p* = 0.0397) (Fig. 1C).

**Table 1:** Demographic and clinical data associated with 112 primary lesions and 17 metastatic lesions.

Parameter	Number (%)
Sex	
Male	69 (61.6)
Female	43 (38.4)
Age (years)	
Range	42–91
Mean	72.8
Primary tumor site	
Genital area	102 (91.1)
Perianal area	5 (4.5)
Axillary area	5 (4.5)
Nodule formation	
Present	18 (16.1)
Absent	94 (83.9)
Ulceration	
Present	48 (42.9)
Absent	64 (57.1)
Tumor invasion level	
In situ	68 (60.7)
≤ 3 mm	34 (30.4)
> 3 mm	10 (8.9)
TNM stage	
I or II	96 (85.7)
III or IV	16 (14.3)
Metastatic site (n = 17)	
Lymph node	15 (88.2)
Skin	2 (11.8)

### FOXM1 expression in an EMPD cell line, KS-EMPD-1

**Figure 2:**
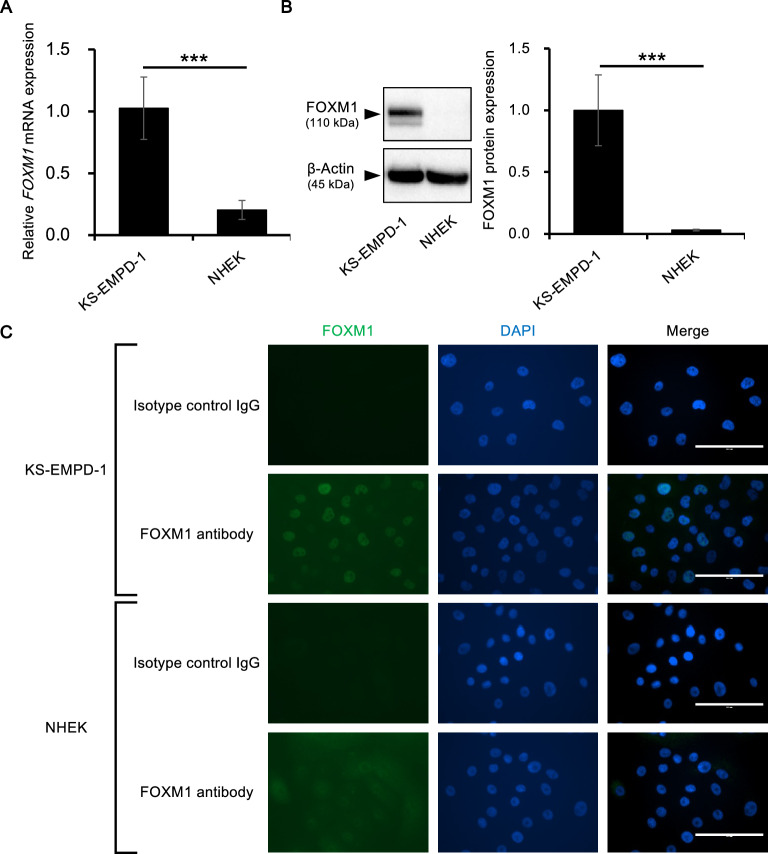
FOXM1 is highly expressed in KS-EMPD-1 cells. (**A**) *FOXM1* mRNA expression in KS-EMPD-1 cells and NHEKs. Mean ± SD of *FOXM1* obtained from three independent experiments is shown. ***p < 0.001. (**B**) FOXM1 protein expression in KS-EMPD-1 cells and NHEKs. Representative blot images (left) and mean ± SD of FOXM1 protein (right) are shown. Experiments were independently repeated three times. Original, uncropped images are shown in Supplementary Fig. S1. ***p < 0.001. (**C**) Immunocytochemical images of FOXM1 in KS-EMPD-1 cells and NHEKs. FOXM1 (green), DAPI (blue, nuclear), and merged images of FOXM1 and DAPI are shown. Scale bars = 100 μm.

Next, we investigated FOXM1 expression in vitro using an EMPD cell line, KS-EMPD-1. Normal epidermal keratinocytes (NHEKs) were also analyzed to determine FOXM1 expression in non-malignant skin cells for comparison. *FOXM1* mRNA was significantly highly expressed in KS-EMPD-1 compared with that in NHEKs (Fig. 2A). In accordance with the findings on mRNA expression, FOXM1 protein was significantly highly expressed in KS-EMPD-1 cells compared with that in NHEKs (Fig. 2B, Supplementary Fig. S1). To further confirm the protein expression and localization, FOXM1 was immunocytochemically stained and observed. As shown in Fig. 2C, FOXM1 was expressed in the nucleus of KS-EMPD-1 cells, but not expressed in NHEKs.

### Thiostrepton, a FOXM1 inhibitor, inhibits proliferation of KS-EMPD-1 cells

**Figure 3:**
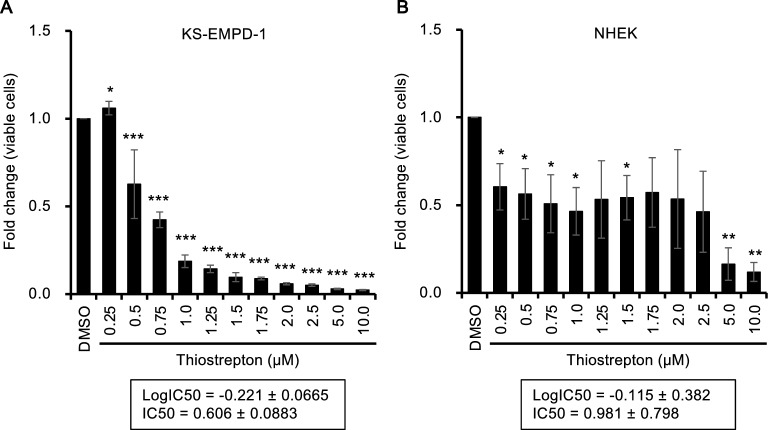
KS-EMPD-1 is sensitive to a FOXM1 inhibitor, thiostrepton. KS-EMPD-1 cells and NHEKs were incubated with DMSO (0.1%) or various concentrations of thiostrepton (0.25–10.0 μM) for 72 h and evaluated for the number of viable cells using a formazan-based method. Mean ± SD of fold change (viable cells) calculated from three independent experiments is shown. The IC_50_ of thiostrepton is shown in the boxes below the graphs. *p < 0.05, **p < 0.01, and ***p < 0.001.

Thiostrepton is an antibiotic that is also known to potently inhibit FOXM1 by directly interacting with it. When the KS-EMPD-1 cells were treated with thiostrepton, it significantly reduced their viability compared with that under the vehicle (dimethyl sulfoxide: DMSO)-treated conditions. The IC_50_ of thiostrepton was calculated as 0.606 ± 0.0883 μM in KS-EMPD-1 cells (Fig. 3A). The effects of thiostrepton on NHEKs (non-malignant skin cells) were also assessed. Thiostrepton treatment significantly decreased the number of NHEKs with IC_50_ of 0.981 ± 0.798 μM (Fig. 3B).

### Effects of FOXM1 knockdown on proliferation of KS-EMPD-1 cells

**Figure 4:**
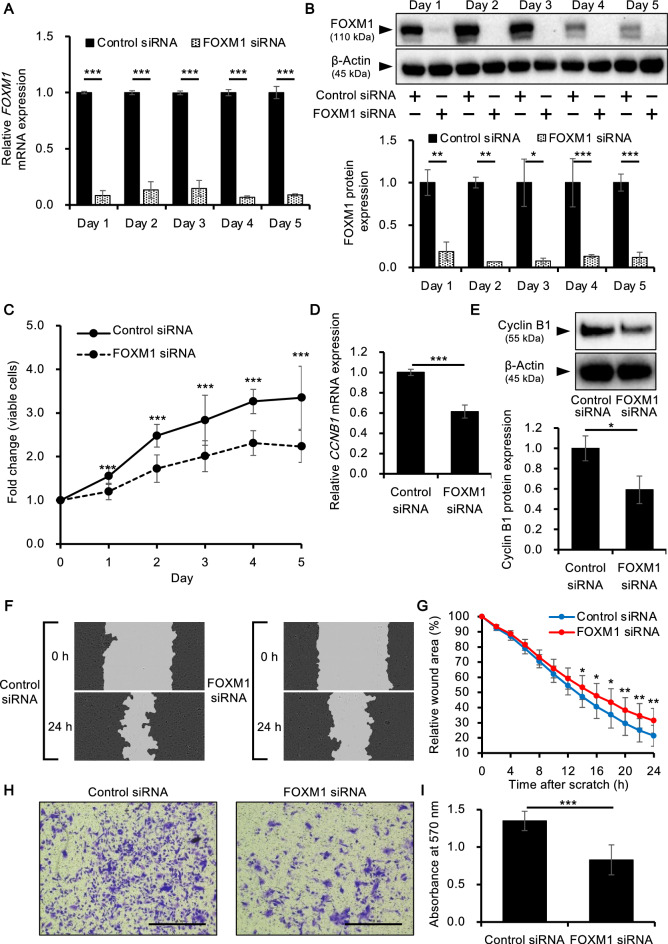
FOXM1 knockdown inhibits proliferation, migration, and invasion of KS-EMPD-1 cells. Cells were transfected with FOXM1 siRNA and evaluated for their proliferation, migration, and invasion. (**A**) Knockdown efficiency of *FOXM1* mRNA. Mean ± SD of *FOXM1* calculated from three independent experiments is shown. ***p < 0.001. (**B**) Knockdown efficiency of FOXM1 protein. Representative blot images (upper) and mean ± SD of FOXM1 protein (lower) are shown. Experiments were independently repeated three times. Original, uncropped images are shown in Supplementary Fig. S2A. *p < 0.05, **p < 0.01, and ***p < 0.001. (**C**) Viable cells in control and FOXM1 siRNA-transfected conditions were quantified using a formazan-based method. Experiments were independently repeated three times. ***p < 0.001. (**D**) *CCNB1* mRNA expression in control or FOXM1 siRNA-transfected cells at 24 h post-transfection. Mean ± SD of *FOXM1* expression calculated from three independent experiments is shown. ***p < 0.001. (**E**) Cyclin B1 protein expression in control or FOXM1 siRNA-transfected cells at 48 h post-transfection. Experiments were independently repeated three times and representative blot images (upper) and mean ± SD of cyclin B1 protein expression (lower) are shown. Original, uncropped images are shown in Supplementary Fig. S2B. *p < 0.05. Cells were transfected with siRNA for 48 h. The cell monolayers were then scratched and the wound area were captured for 24 h. Representative images (**F**) and wound area relative to that at 0 h (**G**) calculated from three independent experiments are shown. *p < 0.05 and **p < 0.01. Cells were transfected with siRNA for 48 h and evaluated for cell invasion. Representative images (**H**) and mean ± SD of absorbance of cell staining dye (**I**) are shown. Experiments were independently repeated three times. Scale bars = 1.0 mm. ***p < 0.001.

The effect of FOXM1 inhibition on cell proliferation was further tested by knocking down FOXM1 using specific small interfering RNA (siRNA). Knockdown efficiency was over 80% at both mRNA (Fig. 4A) and protein levels (Fig. 4B, Supplementary Fig. S2A). Cell proliferation was significantly inhibited in FOXM1 siRNA-transfected cells compared with that of control siRNA-transfected cells (Fig. 4C). The expression of cyclin B1, one of the downstream molecules of FOXM1 regulating the cell cycle, was further assessed in siRNA-transfected cells. The gene expression of cyclin B1 (*CCNB1*) was significantly downregulated in FOXM1 siRNA-transfected cells compared with that in control siRNA-transfected cells (Fig. 4D). Western blotting confirmed that cyclin B1 protein expression was also significantly downregulated in FOXM1-inhibited cells (Fig. 4E, Supplementary Fig. S2B). In addition, knockdown of FOXM1 significantly impaired tumor cell migration (Fig. 4F, G) and invasion (*p < 0.05, **p < 0.01, and ***p < 0.001, Fig. 4H, I).

### Effect of FOXM1 knockdown on chemosensitivity of KS-EMPD-1 cells to anticancer drugs

**Figure 5:**
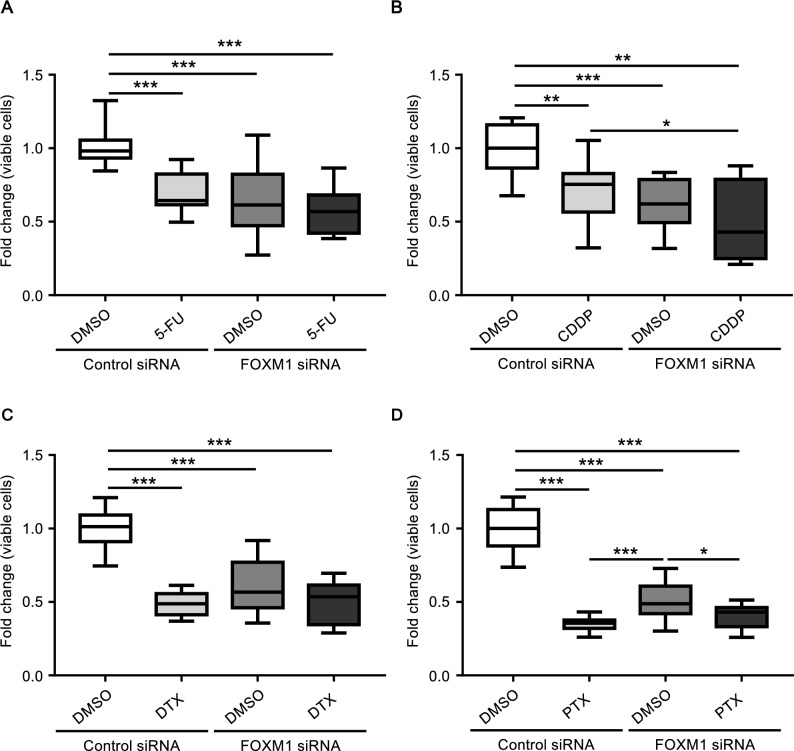
Knockdown of FOXM1 increases the chemosensitivity of KS-EMPD-1 cells. KS-EMPD-1 cells were transfected with control or FOXM1 siRNA for 24 h and further treated with vehicle control or anticancer drugs for 72 h. The viable cells were then quantitated using a formazan-based method. 5-Fluorouracil (5-FU, final concentration 100 nM), docetaxel (DTX, final concentration 5 μM), and paclitaxel (PTX, final concentration 1 μM) were dissolved in DMSO and cisplatin (CDDP, final concentration 10 μM) was dissolved in saline and further added to the culture medium. Concentration of anticancer drugs was determined based on the C_max_ of each drug. Mean ± SD of fold change of viable cells calculated from three independent experiments is shown. *p < 0.05, **p < 0.01, and ***p < 0.001.

To investigate the effects of FOXM1 knockdown on chemosensitivity to anticancer drugs, KS-EMPD-1 cells were transfected with FOXM1 siRNA and further treated with anticancer drugs (5-FU, CDDP, DTX, and PTX) used for treating EMPD. Knockdown of FOXM1 did not affect the chemosensitivity of KS-EMPD-1 cells to 5-FU (Fig. 5A). Knockdown of FOXM1 and further treatment with CDDP significantly decreased the proportion of viable cells compared with the control siRNA-transfected and CDDP -treated condition, but the change was only small (Fig. 5B). Similar to the findings for 5-FU, knockdown of FOXM1 did not affect the chemosensitivity of KS-EMPD-1 cells to DTX and PTX (Fig. 5C, D).

## Discussion

Forkhead Box proteins, also known as Forkhead Homeobox proteins, are a group of transcription factors that belong to the winged helix transcription factors. These transcription factors originated from helix-turn-helix motifs of bacteria and are evolutionarily conserved ^49,61–63^. They were first discovered in the eukaryotic Forkhead Homeotic gene and have been found to have various roles in cell development, maintenance of cardiac equilibrium, angiogenesis, and tumorigenesis of different types of cancer ^49,61–63^. The human FOX group proteins have 19 subclasses (FOX-A to FOX-S) based on sequence conservation, and each subclass has different members ^49,61–63^. Among all FOX family members, FOXM1 is a central tissue factor and plays a key role in embryonic development. Its aberrant expression is associated with the development and progression of various types of cancer ^49^. FOXM1 is responsible for regulating cell proliferation and increasing the expression of genes related to DNA replication, G_1_/S and G_2_/M transitions, cyclin A2, Cdc25A phosphatase, ATF2, and JNK1 ^49,64–66^. Overexpression and gene amplification of FOXM1 are linked to several types of cancer and this association has been found to result in a poor prognosis ^51,52,56–59^. Additional research has shown that FOXM1 plays a role in invasion, migration, angiogenesis, stemness, and therapy resistance, indicating its insidious nature ^62,67–71^.

In this study, we immunostained a relatively large number of EMPD clinical samples and conducted several in vitro analyses using our newly established EMPD cell line, KS-EMPD-1 ^60^, in order to investigate the role of FOXM1 in the tumorigenesis of this disease and examine FOXM1 as a potential therapeutic target of EMPD. We found that, even in the early stages of EMPD, most cases showed strong and abundant positive expression of FOXM1, and its positivity increased with tumor progression. Considering the non-aggressive nature of EMPD, in that it arises in the epithelium of the skin and mucosa and remains indolent there for a long period as an in situ lesion ^2^, the overexpression of FOXM1 in EMPD is interesting since such overexpression is commonly related to aggressiveness in many other cancers ^57–59^. The KS-EMPD-1 cell line strongly expressed FOXM1 compared with NHEKs and knockdown of FOXM1 significantly reduced viable EMPD cells, suggesting its pivotal role in EMPD cell survival. Knockdown of FOXM1 also resulted in the downregulation of Cyclin B1, a molecule crucial for cell cycling. Knockdown of FOXM1 significantly impaired tumor cell migration and invasion. In addition, a potent FOXM1 inhibitor, thiostrepton, strongly impaired tumor cell viability in a dose-dependent manner. A topical ointment containing thiostrepton is currently available on the market for use on animals ^72^, but it is not approved for humans. EMPD is a skin condition that affects the epithelium, which is the outermost layer of the body. In most cases, EMPD cells remain in the epithelium. Therefore, the topical application of thiostrepton could be an effective way of delivering this FOXM1 inhibitor to the tumor cells and alleviating the disease. However, unlike in other cancers in which FOXM1 contributes to chemoresistance ^51,52,58,59^, sufficient improvement of chemosensitivity by FOXM1 inhibition was not observed in the KS-EMPD-1 cells because every cytotoxic agent used in this experiment (5-FU, CDDP, DTX, and PTX) massively reduced viable cell counts.

A major limitation of this study is the limited number of cell lines used. However, until we established the KS-EMPD-1 cell line ^60^, there was no cell line available for EMPD. Currently, KE-EMPD-1 is the only cell line available for EMPD.

In conclusion, our findings indicate a significant increase in the expression of FOXM1 in EMPD. Strong and abundant FOXM1 expression was observed in both early and advanced stages of the disease. Further research is needed to explore the potential of FOXM1 as a therapeutic target for treating both early and advanced EMPD.

## Methods

### Reagents

DMSO (07–4860-5; Sigma-Aldrich, St. Louis, MO) was used as a solvent of chemical compounds. Docetaxel (DTX, 047-31281), paclitaxel (PTX, 163-28163), 5-fluorouracil (5-FU, 068-01401), and cisplatin (CDDP, 033-20091; all purchased from Fujifilm Wako Pure Chemicals, Osaka, Japan) were dissolved in DMSO or saline before being used for the experiments. A potent inhibitor of FOXM1, thiostrepton (T8902; Sigma-Aldrich), was dissolved in DMSO before its use.

### Ethical approval

The current study was carried out following the tenets of the Declaration of Helsinki. Immunohistochemical analysis of patients’ samples was approved by the Ethics Committee of Kyushu University Hospital (approval number 30-363, approved on Nov. 27, 2018). Written informed consent was obtained from each participant before their inclusion in the study.

### Immunohistochemistry (IHC)

FFPE EMPD tissues were obtained from the archives of Kyushu University Hospital. Prior to this study, secondary EMPD had been carefully excluded by clinical examination and a panel of IHC (CK7, CK20, GCDFP15 and CDX2, etc.). FFPE samples were sliced into 4-μm-thick sections and stained for FOXM1 via slightly modified versions of previously reported methods ^73–75^. These sections were incubated with primary antibody (rabbit anti-human FOXM1, 1:1,000, sc-502; Santa Cruz Biotechnology Inc., Dallas, TX) for 90 min at room temperature and further incubated with secondary antibody (N-Histofine Simple Stain AP MULTI, 414261; Nichirei Biosciences, Tokyo, Japan) for 30 min at room temperature. Next, the sections were treated with FastRed II (415261; Nichirei Biosciences) and counterstained with hematoxylin (30002; Muto Pure Chemicals, Tokyo, Japan). For CK7, antigen was retrieved by protease (715231; Nichirei Biosciences) incubation. Then, we used mouse anti-human CK7 (prediluted by supplier, 713481; Nichirei Biosciences) as the primary antibody followed by N-Histofine Simple Stain MAX-PO MULTI (724152; Nichirei Biosciences) as the secondary antibody. The 3,3′-diaminobenzidine tetrahydrochloride (725191; Nichirei Biosciences) was used as a chromogenic substrate. Stained sections were observed and images were captured using a Nikon ECLIPSE 80i microscope (Nikon, Tokyo, Japan).

### Evaluation of FOXM1 IHC staining

The stained samples were independently observed by two dermatologists (T.I. and Y.K.-I.), who were blinded to the patients’ information. Immunoreactivity of FOXM1 was defined as cells showing nuclear staining with or without cytoplasmic staining patterns. Tumors with strong staining intensity in > 10% of the tumor cells were recorded as having positive immunoreactivity, based on a previously reported method ^51,52,56,57^.

### Cell culture

KS-EMPD-1 cells (originally established by Ito et al. ^60^) were cultured in Endothelial Cell Growth Medium 2 Kit (C-22111; Takara Bio Inc., Tokyo, Japan) supplemented with 10 ng/mL Heregulin β1 (100-03; PeproTech, Cranbury, NJ). NHEKs (00192907; Lonza, Basel, Switzerland) were cultured in KGM-Gold Basal Medium with SingleQuots supplements (00192060; Lonza). Medium was refreshed every 2–3 days and cells were passaged at 80% confluence using 0.05 w/v% trypsin/EDTA (202-16931; Fujifilm Wako Pure Chemicals). Contamination of mycoplasma was tested using CycleavePCR Mycoplasma Detection Kit (CY232; Takara Bio Inc.). Cells were confirmed to be mycoplasma free.

### Immunocytochemistry

Immunocytochemistry was performed as described in a previous report ^60^. The antibodies used were rabbit anti-human FOXM1 antibody (1:100, 5436; Cell Signaling Technology, Danvers, MA) as primary antibody and AlexaFluor®488-conjugated goat anti-rabbit IgG (1:400, A11008; Thermo Fisher Scientific, Waltham, MA) as secondary antibody. Stained samples were then covered with Vectashield mounting medium with DAPI (H-1200; Vector Laboratories Inc., Newark, CA). Images were observed and captured using an EVOS FL imaging system (Thermo Fisher Scientific).

### siRNA transfection

siRNA transfection was performed using Lipofectamine RNAiMAX (13778150; Thermo Fisher Scientific), in accordance with the manufacturer’s protocol. Cells were seeded into 6-well plates (2 × 10^5^ cells/well), 12-well plates (1 × 10^5^ cells/well), or 96-well plates (5000 cells/well) and incubated for 24 h at 37 °C in 5% CO_2_. Diluted negative control siRNA (AM4635; Invitrogen, Waltham, MA) or FOXM1 siRNA (s5250; Invitrogen) was mixed with Opti-MEM I reduced serum medium (31985-070; Thermo Fisher Scientific) and RNAiMAX, and incubated for 20 min at room temperature. The final siRNA concentration was set to 10 nM. The resulting siRNA-Lipofectamine complex was added to the cells and further incubated at 37 °C in 5% CO_2_.

### Cell proliferation assay

Cells were seeded into 96-well plates (5,000 cells/well) and transfected with control or FOXM1 siRNAs as described above and were cultured for 1–5 days. Then, the viable cells were quantitated with Cell Counting Kit-8 (CCK-8, 343-07623; Dojindo, Kumamoto, Japan). On each day, 10 μL of CCK-8 solution was added to each well and the culture plate was incubated for 2–4 h at 37 °C. After the incubation, absorbance at 450 nm was measured using an iMark microplate reader (Bio-Rad Laboratories, Inc., Hercules, CA).

### RNA extraction and quantitative reverse-transcription polymerase chain reaction (qRT-PCR)

RNA extraction, cDNA synthesis, and following qRT-PCR were performed as described in a previous report ^60^. β-Actin (*ACTB*) was used as a housekeeping gene. The sequences of the primers were as follows: *FOXM1*, forward 5′-TCTGCCAATGGCAAGGTCTCCT-3′, reverse 5′-CTGGATTCGGTCGTTTCTGCTG-3′; *cyclin B1* (*CCNB1)*, forward 5′-GACCTGTGTCAGGCTTTCTCTG-3′, reverse 5′-GGTATTTTGGTCTGACTGCTTGC-3′; and *ACTB*, forward 5′-ATTGCCGACAGGATGCAGA-3′, reverse 5′-GAGTACTTGCGCTCAGGAGGA-3′.

### Protein extraction and western blotting

Western blotting was performed as described in previous publications ^76–78^. Antibodies used were rabbit anti-human FOXM1 (1:1000, 5436; Cell Signaling Technology), rabbit anti-human cyclin B1 (1:1000, 12231, Cell Signaling Technology), and rabbit anti-human β-actin (1:2000, 4970; Cell Signaling Technology). Resultant immunological bands were observed using the ChemiDoc XRS Plus System (Bio-Rad Laboratories). The intensity of the bands was quantified using ImageJ software (National Institutes of Health, Bethesda, MD). Uncropped, full-length original gel images are shown in Supplementary Figs. S1 and S2.

### Invasion assay

The invasion of control or FOXM1 siRNA-transfected cells was evaluated with a CytoSelect 24-well cell invasion assay (CBA-110; Cell Biolabs Inc., San Diego, CA). Cells were transfected with siRNA as explained above and at 48 h post-transfection, cells were harvested and used for the invasion assay. Invasion assay was performed evaluated as reported previously ^60^.

### Migration assay

Cell migration assay was performed as described in a previous report with slight modifications ^78^. Cells were seeded into a 96-well ImageLock tissue culture microplate (1.5 × 10^4^ cells/well; Essen Bioscience, Ann Arbor, MI) pre-coated with type I collagen (Nitta Gelatin Inc., Osaka, Japan) and incubated at 37 °C in 5% CO_2_. Twenty-four hours after the incubation, cells were transfected with siRNAs as described above. At 48 h, cell monolayers were scratched with a wound-maker (Essen Bioscience) and images of the scratched area were automatically captured every 2 h for a total of 24 h in the IncuCyte Cell Imaging System (Essen Bioscience). The wound area relative to that at 0 h was calculated by IncuCyte software (Essen Bioscience).

### *Thiostrepton treatment and IC*_*50*_* measurement*

Cells were seeded into 96-well plates (5,000 cells/well) and incubated at 37 °C in 5% CO_2_. At 24 h, the medium was replaced with medium containing vehicle (DMSO, 0.1%) or various concentrations of thiostrepton (0.25–10.0 μM) and the cells were further incubated for 72 h at 37 °C in 5% CO_2_. The range of thiostrepton concentrations was determined based on previous publications with slight modifications ^51,52^. After the incubation, viable cells were quantified using CCK-8 solution as described above. The IC_50_ of thiostrepton was calculated using GraphPad Prism 7 software (GraphPad Software, San Diego, CA).

### Chemosensitivity assay

The chemosensitivity of FOXM1-knockdown cells to 5-FU, CDDP, DTX, and PTX was tested. Cells were transfected with negative control siRNA or FOXM1 siRNA as described above. Twenty-four hours post-transfection, cells were incubated with the anticancer drugs at 37 °C in 5% CO_2_. DMSO was used as a solvent of 5-FU, DTX, and PTX, while saline (0.9% sodium chloride solution) was used as a solvent of CDDP. The concentrations of the drugs were determined considering the C_max_ of each drug (100 nM, 10 μM, 5 μM, and 1 μM for 5-FU, CDDP, DTX, and PTX, respectively) ^60,79^. At 72 h of incubation, viable cells were quantified using CCK-8 solution as described above.

### Statistical analysis

All experiments were repeated independently at least three times and the quantitative results are shown as mean ± standard deviation (SD). Statistical analysis was performed using GraphPad Prism 7 software. The normality of the obtained data was investigated using the Shapiro–Wilk test and the significance of differences between two groups was tested by Student’s unpaired two-tailed t-test. If the data were not normally distributed, the Mann–Whitney U test was used. To analyze the significance of differences among three or more groups, one-way ANOVA and subsequent multiple comparison test were performed. The Kaplan–Meier method and the log-rank test were used to evaluate disease-specific survival. Disease-specific survival was calculated from the date of the diagnosis to the date of death due to EMPD. Data on patients without death were censored on the date of the last visit. Data on patients who had died of other causes were censored at the time of death. In the survival analysis, patients were stratified by FOXM1 expression of primary tumor. *p* < 0.05 was defined as statistically significant.

### Supplementary Information

Supplementary Figures.

## Data Availability

All data obtained or analyzed during this study are included in the main text and figures.
