# Supplementary material for: FOXM1: a new therapeutic target of extramammary Paget disease
Source: Sci Rep. 2024 Feb 19;14:4048. doi: 10.1038/s41598-024-54773-8 (PMC10876583; doi:10.1038/s41598-024-54773-8)
Supplement: Supplementary file 1 — Supplementary Figures. [file 41598_2024_54773_MOESM1_ESM.docx]

**FOXM1: a new therapeutic target of extramammary Paget’s disease**

Takamichi Ito^1*^, Yuka Tanaka^1^, Yumiko Kaku-Ito^1^, Yoshinao Oda^2^, Takeshi Nakahara^1^

^1^Department of Dermatology, Graduate School of Medical Sciences, Kyushu University, Fukuoka 812-8582, Japan

^2^Department of Anatomic Pathology, Graduate School of Medical Sciences, Kyushu University, Fukuoka 812-8582, Japan

*Correspondence to: Takamichi Ito, M.D., Ph.D.

Department of Dermatology, Graduate School of Medical Sciences, Kyushu University

3-1-1 Maidashi, Higashi-ku, Fukuoka 812-8582, Japan

Tel.: +81-92-642-5585

Fax: +81-92-642-5600

E-mail: ito.takamichi.657@m.kyushu-u.ac.jp

This file contains:

Supplementary Figure S1

Supplementary Figure S2


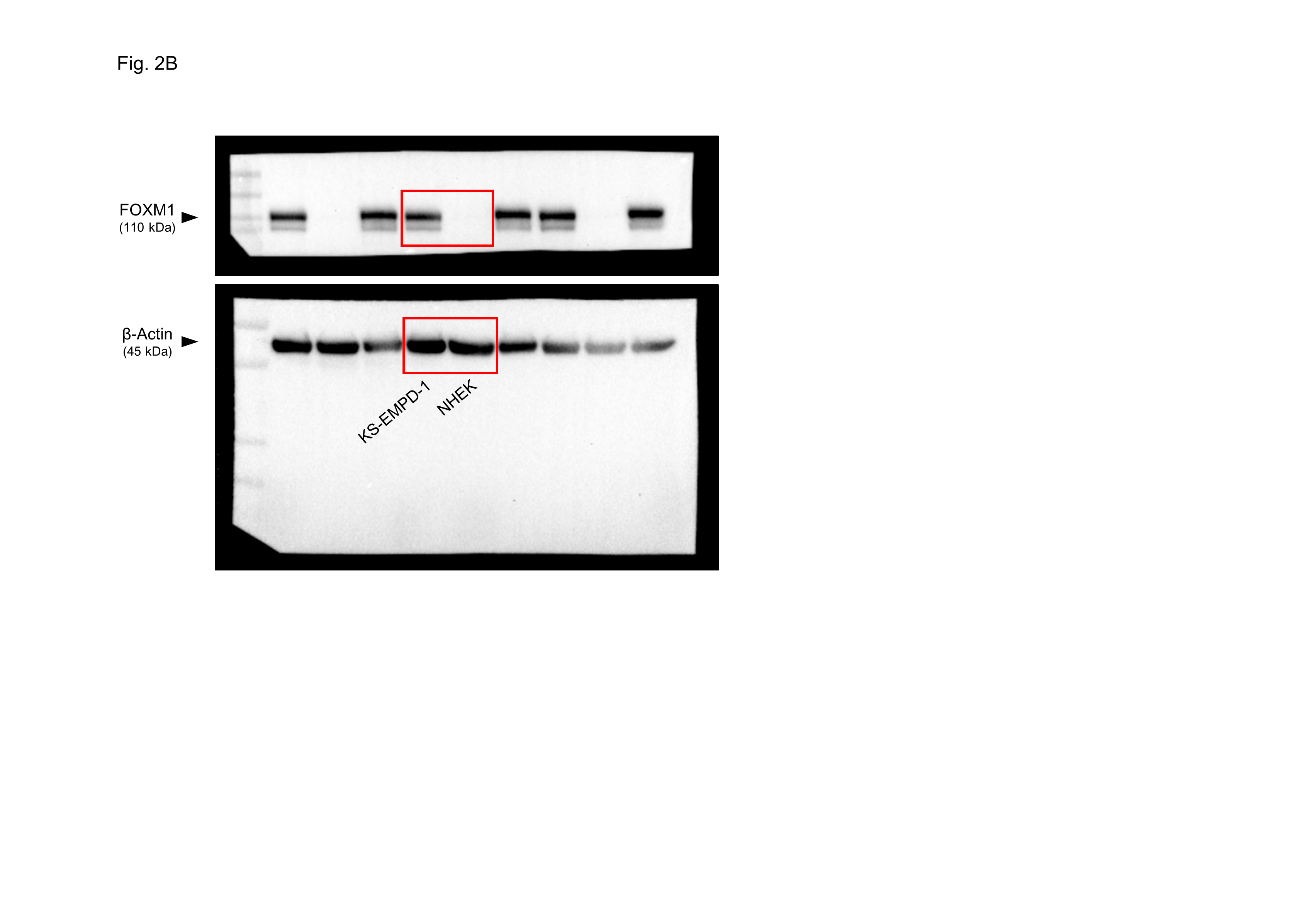


**Supplementary Figure S1. Full-length blots presented in Fig. 2B.** Protein expression of FOXM1 and β-actin in KS-EMPD-1 was determined by western blotting. Unedited original images of blots are shown. The signal for each protein was analysed using ImageJ software and was normalized against that of β-actin. Membranes were cut based on the size marker and hybridized with different kinds of antibodies when needed. The red boxes indicate the croped areas shown in Fig. 2B.


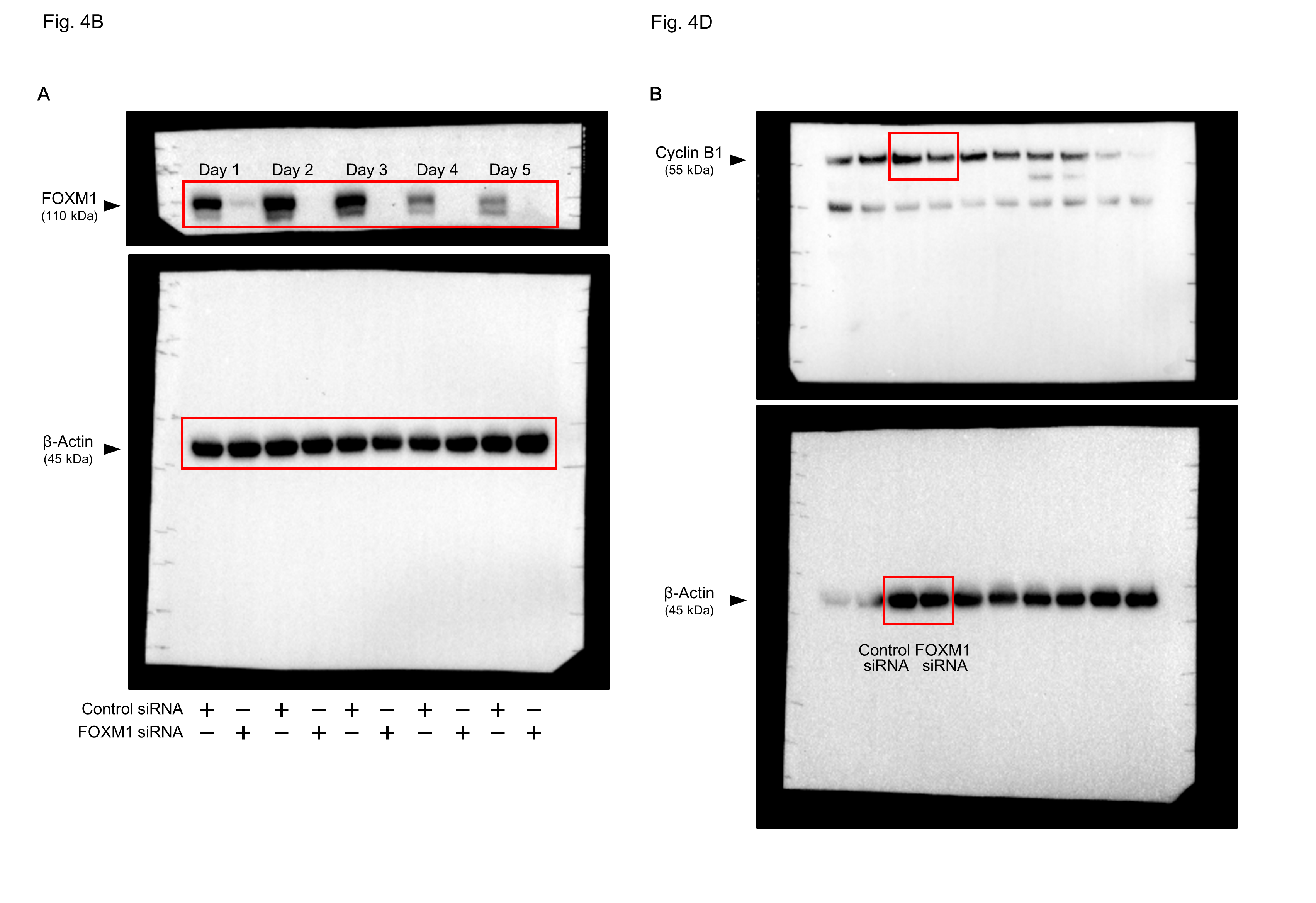


**Supplementary Figure S2. Full-length blots presented in Fig. 4B and 4E.** Protein expression of (**A**) FOXM1 and β-actin, and (**B**) cyclin B1 and β-actin in FOXM1-inhibited KS-EMPD-1 was determined by western blotting. Unedited original images of blots are shown. The signal for each protein was analysed using ImageJ software and was normalized against that of β-actin. Membranes were cut based on the size marker and hybridized with different kinds of antibodies when needed. The red boxes indicate the croped areas shown in Fig. 4B and 4E.
